# Observational Study of Men and Women with Breast Cancer in Terms of Overall Survival

**DOI:** 10.3390/cancers16173049

**Published:** 2024-09-01

**Authors:** Vlad Bogdan Varzaru, Diana-Maria Anastasiu-Popov, Anca-Elena Eftenoiu, Roxana Popescu, Daliborca Cristina Vlad, Cristian Sebastian Vlad, Aurica Elisabeta Moatar, Daniela Puscasiu, Ionut Marcel Cobec

**Affiliations:** 1 Doctoral School, Faculty of Medicine, “Victor Babes” University of Medicine and Pharmacy Timisoara, 300041 Timisoara, Romania; 2 ANAPATMOL Research Center, Faculty of Medicine, “Victor Babes” University of Medicine and Pharmacy Timisoara, Eftimie Murgu Square No. 2, 300041 Timisoara, Romania; 3 Clinic of Obstetrics and Gynecology, Diakoneo Diak Klinikum, 74523 Schwäbisch Hall, Germany; 4 Department of Medical Genetics, “Carol Davila” University of Medicine and Pharmacy, 014461 Bucharest, Romania; 5 Department of Cell and Molecular Biology, “Victor Babes” University of Medicine and Pharmacy Timisoara, 300041 Timisoara, Romania; 6 Department of Pharmacology, “Victor Babes” University of Medicine and Pharmacy Timisoara, 300041 Timisoara, Romania; 7 Clinic of Internal Medicine-Cardiology, Klinikum Freudenstadt, 72250 Freudenstadt, Germany; 8 Clinic of Obstetrics and Gynecology, Klinikum Freudenstadt, 72250 Freudenstadt, Germany

**Keywords:** breast cancer, male breast cancer, survival

## Abstract

**Simple Summary:**

Breast cancer is the most common cancer and the leading cause of cancer-related deaths among women globally, with over 2.26 million new cases in women in 2020. Male breast cancer, while rare (less than 1% of cases), is treated similarly to female breast cancer despite some clinical differences. Advances in treatment, such as using genomic and immunohistochemical markers, have significantly improved survival rates for women. Men with breast cancer are often older at diagnosis, and their prognosis may differ due to age and other factors. The survival analysis conducted on 2162 patients (19 males and 2143 females) suggests that male breast cancer may have a poorer prognosis compared to female breast cancer.

**Abstract:**

Breast cancer is one of the most common cancers and the leading cause of cancer death in women. Less than 1% of breast cancer cases are male breast cancers. Although there has been significant progress made in the management of breast cancer, due to its rarity among men, the question of whether men and women with breast cancer have the same treatment response and survival rate still needs to be answered. The primary goal of this study is to compare survival outcomes between male and female breast cancer patients. Material and Method: This cohort study represents a retrospective and anonymized data analysis of 2162 breast cancer cases (19 males and 2143 females), registered over a period of 12 years, from 1 January 2010 to 31 December 2021, in the Clinic of Obstetrics and Gynecology, Diakoneo Diak Klinikum Schwäbisch Hall, Germany. Results: According to the Kaplan–Meier survival analysis, the estimated overall 3-year survival rate was 91.1% for women and 88.9% for men. The log-rank test of equality of survival distributions indicated a statistically significant difference in survival times between the two groups (*p* = 0.009). In the subsequent age-matched Kaplan–Meier analysis, the *p*-value was below the significance threshold (*p* = 0.068). Conclusions: Male breast cancer is a rare disease that may show some particularities in terms of survival compared to female breast cancer.

## 1. Introduction

The World Health Organization (WHO) states that breast cancer is the most commonly diagnosed cancer in women and the leading cause of cancer-related deaths among women all over the world [1]. Male breast cancer accounts for less than 1% of all breast cancer cases [2,3]. In 2022, the incidence of breast cancer in women exceeded 2.29 million [4]. 

This rare disease in men is treated in a manner similar to the therapy standards for women, although the clinicopathological particularities of male breast cancer, according to some authors, differ from those of female breast cancer [5,6].

Male breast cancer patients present risk factor particularities, such as age, family history, Jewish descent, testicular disease, non-cancerous breast conditions, Klinefelter syndrome, and BRCA2 mutations, while prognostic factors and survival are considered to be the same as in women [7].

The diagnosis is an important step in the management of breast cancer. Advances in medical imaging have become not only indispensable in the early diagnosis of breast cancer but also a reliable component in patient evaluation during therapy. Routine medical examinations play a key role in reducing the mortality rate of breast carcinoma [8].

The advances in the treatment management of female breast cancer over the years can be clearly seen in the therapies available nowadays, from the simple radical mastectomy in the past to a now complex and well-coordinated interdisciplinary therapy that uses genomic markers such as BRCA1, BRCA2, and PIK3CA; immunohistochemical markers like estrogen receptors (ER), progesterone receptors (PR), and human epidermal growth factor receptor 2 (Her2); and immunomarkers like PD-L1 and tumor-infiltrating lymphocytes to determine the most suitable treatment for the patients and greatly improve their overall success rates [9,10].

It is estimated that about 5% to 10% of breast cancer cases are hereditary [11]. Germline pathogenic (or likely pathogenic) variants in BRCA genes are the primary genetic risk factors, with BRCA1 mainly associated with female breast cancer and BRCA2 with male breast cancer [12,13]. Genetic testing for BRCA1 and BRCA2 mutations is generally recommended for individuals with a family or personal history of cancer [14].

The standard surgical treatment for breast cancer includes total mastectomy or breast-conserving surgery, though the latter is rarely performed in men due to the high risk of recurrence [15,16]. Sentinel lymph node biopsy and axillary lymph node dissection are performed based on clinical guidelines for managing the axilla in breast cancer patients, which have been progressively updated. Anatomical differences between sexes must be considered in surgical management [16,17]. Breast-conserving surgery is indicated for non-invasive breast cancer, invasive carcinomas with a favorable tumor-to-breast volume ratio, and invasive carcinomas with intraductal components, provided that resection margins are clean [18].

The current standard breast cancer management in women includes neoadjuvant chemotherapy, which often involves targeted agents, and conservative surgery, followed by adjuvant radiotherapy with or without adjuvant chemotherapy and/or endocrine therapy. Adjuvant radiotherapy reduces the rate of local recurrence and, consequently, disease-specific mortality [19].

Therapy guidelines recommend that men receive treatment similar to that provided to women. Those recommendations cover endocrine therapy and germline genetic testing. Some medical societies developed recommendations for the management of male breast cancer [2,20,21]. Male breast cancer is more likely to respond to hormonal therapy because it is more frequently hormone receptor-positive [7].

The quality of life and life expectancy of many patients have significantly improved as a result of research advancements and newly developed therapies in the treatment of breast cancer [22,23]. Some studies show that early identification of female breast cancer not only allows patients to benefit from breast-conserving surgery but also results in a 5-year survival rate of over 90% [24]. The earlier a cancer is detected, the greater the chances of survival and the more significant the treatment success. However, due to its rarity among men, the question of whether men and women with breast cancer have the same treatment response and survival rate still needs to be answered in order to optimize the clinical management of male breast cancer. The primary goal of this study is to compare survival outcomes between male and female breast cancer patients.

## 2. Materials and Methods

This cohort study consists of a retrospective of anonymized breast cancer cases recorded over a period of 12 years, from 1 January 2010 to 31 December 2021, in the Clinic of Obstetrics and Gynecology, Diakoneo Diak Klinikum Schwäbisch Hall, Germany. We included both male and female patients who received a primary breast cancer diagnosis during the above-mentioned period. Patients who received a diagnosis of primary breast cancer during the evaluated period on both sides were included as bilateral and, for the analysis of the histological characteristics, we used the data of whichever tumor was diagnosed first. Exclusion criteria were represented by breast carcinoma in situ (all pertaining to female cases), relapse, or metastasis where the primary tumor was diagnosed prior to the studied period and the absence of a follow-up period of a minimum of one day. During the studied period, we registered a total of 2787 cases. The final cohort consisted of 2162 patients, consisting of 19 males and 2143 females.

All the patients included in our study received therapy and disease management according to the German Guidelines for breast cancer which included surgery, radiotherapy, chemotherapy, and endocrine therapy [18].

Data on demographics, clinical features, histology, and follow-up were retrieved from our database. Data analysis and visualization were performed using IBM SPSS Statistics 26 software. The follow-up period was defined as the time interval between the initial diagnosis and the date of the most recent consultation at our clinic. The Shapiro–Wilk test was used to assess the normality of age at diagnosis, revealing significant deviations from a normal distribution in both groups as well as in the entire dataset. Therefore, a Mann–Whitney U test was conducted to compare age at diagnosis between the two groups. The Kaplan–Meier method was used to estimate overall survival and cases lost to follow-up were censored. Male cases were matched 1:1 to female controls by exact age at diagnosis, selected in random order using the Case Control Matching feature in IBM SPSS Statistics 26. We applied a significance threshold of α = 0.05, corresponding to a 95% confidence interval.

## 3. Results

### 3.1. Descriptive Analysis

Of the 2162 patients included in the analysis, 0.9% (19 cases) were males and 99.1% (2143 cases) were females. In the studied group we registered a total of 243 deaths of all causes (11.2%). Five deaths were attributed to men, representing 2.1% of all deaths. A total of 83 cases (3.8%) were affected bilaterally, of which all were women. 

The youngest patient was 24 years old and the oldest patient was 98 years old, while the median age was 63 (interquartile range: 53–74 years) (SD = 13.559). A Mann–Whitney U test was conducted to compare the ages between male and female breast cancer cases. The results indicate that males (median = 71 years) were significantly older at diagnosis than females (median = 63 years), U = 13,289.5, *p* = 0.009.

The histopathological types of breast cancer registered in our study are presented in Table 1.

There was no case of triple-negative male breast cancer registered. The particularities of the tumor biology of the patients included in our study are described in Table 2.

### 3.2. Survival Analysis

A Kaplan–Meier survival analysis was performed including all-cause deaths. The time was measured in months, calculated as the number of days divided by 365.24/12. Estimations for mean survival times are limited to the largest follow-up time registered in each group, 100.64 months for the male group and 151.03 months for the female group. Median follow-up was 32.86 months for women and 17.28 months for men.

The estimated mean survival times of the performed survival analysis were 67.666, 95% CI [48.432, 86.900] for male patients and 122.043, 95% CI [118.256, 125.830] for female patients. The log-rank test of equality of survival distributions indicated a statistically significant difference in survival times between the two groups (*p* = 0.009). Median survival time was estimated only for the male group: 57.694, 95% CI [40.062, 75.326]. The cumulative survival was greater than 0.5 in the female group; therefore, the median survival time for women could not be estimated.

The overall (all-cause) survival rates estimated by our survival analysis for female patients were 0.964 (SE = 0.004, 95% CI [0.956, 0.972]) for 1 year, 0.911 (SE = 0.007, 95% CI [0.897, 0.926]) for 3 years, and 0.854 (SE = 0.010, 95% CI [0.833, 0.874]) for 5 years. No death was registered during the first year in the male group. The estimated all-cause survival rates in men were 0.889 (SE = 0.105, 95% CI [0.684, 1.000]) for 3 years and 0.400 (SE = 0.174, 95% CI [0.059, 0.741]) for 5 years. The 95% CIs for the survival rates were computed as 1.96 times the standard error in each direction and, when needed, trimmed at the limits.

**Figure 1:**
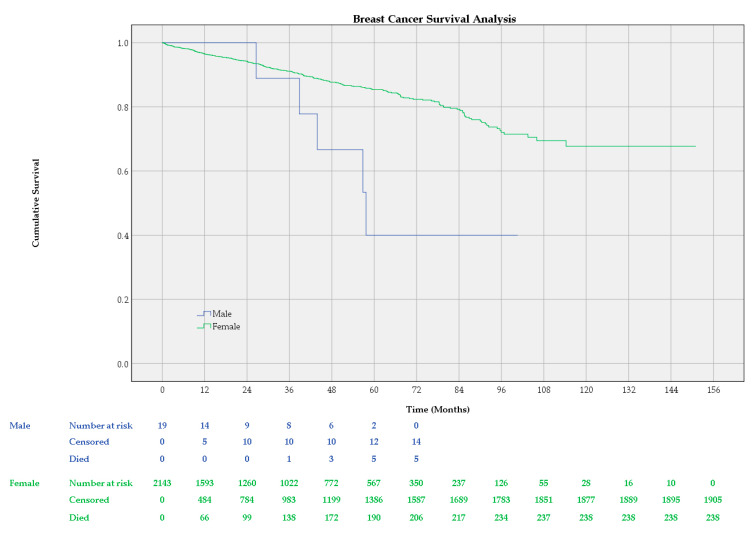
Kaplan–Meier curve of overall survival and number at risk table for male and female breast cancer patients.

Figure 1 shows Kaplan–Meier curves for the cumulative survival of all-cause death among male and female breast cancer patients, as well as the number of cases that have been censored due to loss to follow-up, the number of deaths registered, and the remaining number of patients after intervals of 12 months.

**Figure 2:**
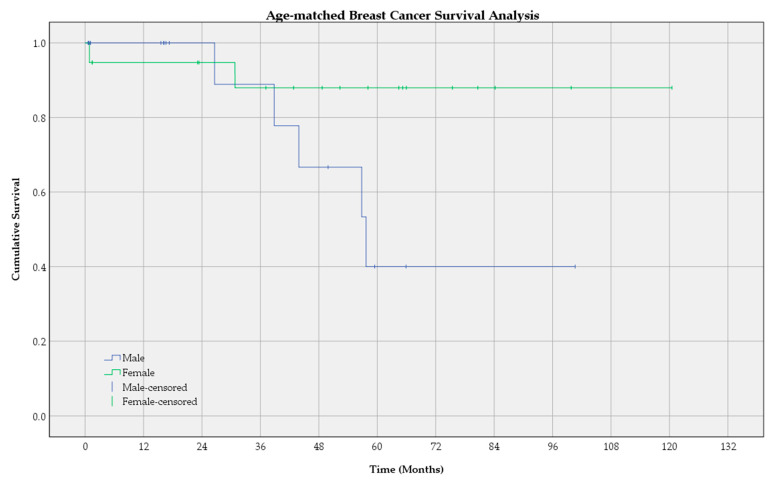
Kaplan–Meier curve of overall survival for age-matched male and female breast cancer patients.

A subsequent Kaplan–Meier survival analysis was carried out on the age-matched dataset of 19 male and 19 female patients (Figure 2). The log-rank test for equality of survival distributions revealed no statistically significant difference in survival times between the two groups (*p* = 0.068).

## 4. Discussion

Although there has been significant progress in the treatment of breast cancer, there are still unanswered questions when it comes to male versus female breast cancer. 

The present study follows patients who received the standard therapy for breast cancer according to the actual medical guidelines which include four main therapy options such as surgery, radiotherapy, chemotherapy, and endocrine therapy [25].

Only 0.9% of the breast cancer patients in the current cohort were men. Less than 1% of breast cancers occur in men, according to the literature [2,3]. Less than 0.2%cancers in men are the breast cancers [25]. 

Our study registered 3.8% cases of bilateral breast cancer, all of which were women, while there was no case of bilateral involvement in men. According to the literature, bilateral involvement is more common in nulligravida women and women who have delivered up to two children, but the longer the interval between the diagnoses of the two cancers, the better the survival rate [26].

In our studied group, we registered a median age at diagnosis of 71 years for men and 63 years for women. This aligns with the existing literature, which reports a median diagnosis age of 68 years for men and 62 years for women [27,28]. Men were significantly older at diagnosis and this fact could contribute to the differences in terms of overall survival observed in the current study, as older age at diagnosis is often associated with comorbidities and reduced physiological resilience [29].

In our study, invasive ductal breast carcinoma was the most prevalent histological type of breast cancer in both men and women, representing 94.7% of male breast cancer and 79.9% of female breast cancer. The histologic types of breast carcinomas that predominate in men are likely of ductal origin, and male breast cancer is mainly composed of ducts and often contains no lobules [30].

The tumor biology of male breast cancer in our study showed a higher prevalence of ER-positive tumors compared to female breast cancer. Specifically, 94.7% of male breast cancers were ER-positive, whereas 82.3% of female breast cancers exhibited the same characteristic. This finding is consistent with prior studies suggesting that male breast cancer is more frequently hormone receptor-positive [25]. The high hormone receptor positivity suggests that hormonal therapies, such as tamoxifen, may be particularly effective in treating male breast cancer [31]. 

In contrast, Her2 positivity was less common in both male and female breast cancers in our cohort. Only 15.8% of male and 14.9% of female breast cancers were Her2-positive. This aligns with the literature indicating that Her2 overexpression is less frequent in male breast cancer [32]. Consequently, targeted therapies like trastuzumab may have limited applicability in male breast cancer treatment.

According to some studies, men had slightly better or equivalent disease-specific survival compared to women [25,33,34,35]. Regarding overall survival, some researchers report lower overall survival rates in men compared to women [36,37,38], while others report no difference between male and female breast cancer [39]. A recent population-based cohort study of 364 039 patients reported significantly different 5-year overall survivals between male and female breast cancer, at 73.9% and 86%, respectively [40]. In a large study involving over x million breast cancer patients, Wang et al. reported 3-year overall survival rates of 86.4% for men and 91.7% for women and 5-year overall survival rates of 77.6% for men and 86.4% for women [36]. The differences in overall survival remained significant, even after adjusting for age, ethnicity, clinical and treatment characteristics, and access to care. Some studies report that male breast cancer has a higher mortality when compared to stage- and subtype-matched female breast cancer [41,42]. 

The survival analysis performed indicated a statistically significant difference between male and female breast cancer survival. Following the Kaplan–Meier curve of male and female breast cancer patients in Figure 1, we notice that men have a better or comparable survival to women in the first three years. The 1-year overall survival was 96.4% for women, while no death was registered in the first year after diagnosis among male patients. The 3-year overall survival was slightly higher in the female group, at 91.1% compared to 88.9% in the male group. The 5-year overall survival in men drops to 40%, while the 5-year overall survival in women gradually decreases to 85.4%. It is worth noting that the estimates for female breast cancer represent better approximations with lower variability since the confidence intervals are tighter than those of male breast cancer estimates. Wide confidence intervals in estimates for male breast cancer reflect greater uncertainty in the results. The median survival time was 57.694 months for men, equating to 4.81 years. Meanwhile, the median survival time for women could not be estimated since the cumulative survival was greater than 0.5 at the end of the follow-up period, which means that the median survival time for women is greater than the largest follow-up time in this group, 151.03 months. The Kaplan–Meier analysis indicates that male breast cancer may have a poorer prognosis compared to female breast cancer. The subsequent age-matched Kaplan–Meier analysis did not meet the significance threshold of 0.05. However, the *p*-value of 0.05 to 0.10 indicates a trend toward statistical significance, suggesting a possible difference that may be obscured by this study’s limitations.

In male breast cancer survivors, the strategies for optimal surveillance are still uncertain [31]. Men with breast cancer face many challenges starting from diagnosis and continuing through the treatment process [43]. Due to the absence of prevention programs for men, low awareness, and lack of information, men usually present at a higher stage of breast cancer disease than women [44]. The differences in survival metrics may vary by country due to differences in access to advanced therapeutic options [45,46,47,48]. Special attention should be given to the disparities between male and female breast cancer and the gap in the overall improvement in survival rates [45]. 

The treatment of male breast cancer largely follows protocols established for female breast cancer, including surgery, radiotherapy, chemotherapy, and endocrine therapy. Our study highlights the need for tailored treatment strategies, considering the specific biological characteristics and later diagnosis stage in men. For instance, the high prevalence of hormone receptor-positive tumors in men underscores the importance of hormonal therapies. However, the absence of triple-negative cases in our male cohort suggests that certain aggressive subtypes are less common, potentially influencing treatment approaches [49]. Despite following similar treatment guidelines, the outcomes differ, indicating a potential gap in the effectiveness of these therapies in men. This gap could be addressed by developing gender-specific guidelines that consider the unique aspects of male breast cancer, such as its hormonal sensitivity and later stage at diagnosis.

The rarity of male breast cancer results in less focused research and awareness and could contribute to later diagnoses, possibly affecting survival rates. Male breast cancer is often diagnosed at a more advanced stage than female breast cancer [25]. Increasing awareness is crucial for early detection and effective treatment. Public health initiatives should aim to educate both the general public and healthcare professionals about the signs and risks of male breast cancer to facilitate earlier diagnosis.

Men with breast cancer may benefit from more aggressive and earlier interventions. Advancements in treatment for female breast cancer, including the use of genomic and immunohistochemical markers, have greatly improved survival rates [50,51]. The significant progress in female breast cancer treatment offers a model, but specific strategies for men need to be developed. It would be beneficial to explore whether men and women with breast cancer have the same treatment response and survival rate when matched for stage and subtype. Further research should also investigate the molecular and genetic differences between male and female breast cancers to identify potential targets for therapy. Given the significant advancements in female breast cancer treatment, similar progress must be made for male breast cancer, through tailored treatment protocols.

### Limitations

A major limitation of the current study is the small number of male breast cancer cases and, consequently, matched cases. Additionally, censoring cases lost to follow-up may result in inaccurate estimates of overall survival. Other limitations that impact the generalizability of the results is bias by confounding factors. These factors could affect the reliability of the findings and the accuracy of the prognosis estimates. Studies on larger cohorts or prospective studies are necessary to further analyze the differences between male and female breast cancer in terms of survival. It would be beneficial to consider the impact of more potential confounding variables, including age at diagnosis, in a multivariate survival analysis.

## 5. Conclusions

Male breast cancer is a rare disease that may show some particularities in terms of survival compared to its female counterpart. The retrospective analysis of 2162 patients in the current study underlines the necessity for increased awareness, early detection, and tailored treatment protocols for male breast cancer. Further research should address the limitations of the current study. 

## Figures and Tables

**Table 1:** Histopathological types of breast cancer registered in our study.

Histopathological Type of Breast Cancer	Total (*n* = 2162)	Female (*n* = 2143)	Male (*n* = 19)
Invasive ductal carcinoma	1727 (79.9%)	1709 (79.7%)	18 (94.7%)
Invasive lobular carcinoma	314 (14.5%)	314 (14.7%)	-
Mucinous carcinoma	45 (2.1%)	45 (2.1%)	-
Tubular carcinoma	23 (1.1%)	23 (1.1%)	-
Papillary carcinoma	13 (0.6%)	13 (0.6%)	-
Breast carcinoma with neuroendocrine differentiation	10 (0.5%)	10 (0.5%)	-
Medullary carcinoma	8 (0.4%)	8 (0.4%)	-
Metaplastic breast cancer	7 (0.3%)	7 (0.3%)	-
Intracystic breast carcinoma	6 (0.3%)	5 (0.2%)	1 (5.3%)
Malign Phyllodes tumor	4 (0.2%)	4 (0.2%)	-
Cribriform breast cancer	2 (0.1%)	2 (0.1%)	-
Mammary Paget disease	2 (0.1%)	2 (0.1%)	-
Breast angiosarcoma	1 (<0.1%)	1 (<0.1%)	-

**Table 2:** Tumor biology characteristics of patients included in this study.

	ER			PR			Her2		
	Total (N = 2162)	Male (N = 19)	Female (N = 2143)	Total (N = 2162)	Male (N = 19)	Female (N = 2143)	Total (N = 2162)	Male (N = 19)	Female (N = 2143)
Negative	353 (16.3%)	1 (2.3%)	352 (16.4%)	552 (25.5%)	4 (21.1%)	548 (25.6%)	1667 (77.1%)	13 (68.4%)	1654 (77.2%)
Positive	1779 (82.3%)	18 (94.7%)	1761 (82.2%)	1579 (73.0%)	15 (78.9%)	1564 (73.0%)	323 (14.9%)	3 (15.8%)	320 (14.9%)
Unknown	30 (1.4%)	-	30 (1.4%)	31 (1.4%)	-	31 (1.4%)	172 (8.0%)	3 (15.8%)	169 (7.9%)

Abbreviations: ER = estrogen receptor, PR = progesterone receptor, Her2 = human epidermal growth factor receptor 2.

## Data Availability

Further information concerning the present study is available from the corresponding author upon reasonable request.
